# Toll-like receptor activation enhances cell-mediated immunity induced by an antibody vaccine targeting human dendritic cells

**DOI:** 10.1186/1479-5876-5-5

**Published:** 2007-01-25

**Authors:** Venky Ramakrishna, John P Vasilakos, Joseph D Tario, Marc A Berger, Paul K Wallace, Tibor Keler

**Affiliations:** 1 Celldex Therapeutics, Inc., Phillipsburg, NJ 08865, USA; 2 3M Pharmaceuticals, St. Paul, MN 55144, USA; 3 Roswell Park Cancer Institute, Buffalo, NY 14263, USA

## Abstract

Previously, we have successfully targeted the mannose receptor (MR) expressed on monocyte-derived dendritic cells (DCs) using a fully human MR-specific antibody, B11, as a vehicle to deliver whole protein tumor antigens such as the human chorionic gonadotropin hormone (hCGβ). Since MRs play a role in bridging innate immunity with adaptive immunity we have explored several toll-like receptor (TLR)-specific ligands that may synergize with MR targeting and be applicable as adjuvants in the clinic. We demonstrate that antigen-specific helper and cytolytic T cells from both healthy donors and cancer patients were effectively primed with B11-hCGβ-treated autologous DCs when a combination of one or several TLR ligands is used. Specifically, concomitant signaling of DCs via TLR3 with dsRNA (poly I:C) and DC TLR 7/8 with Resiquimod (R-848), respectively, elicited efficient antigen presentation-mediated by MR-targeting. We demonstrate that MR and TLRs contribute towards maturation and activation of DCs by a mechanism that may be driven by a combination of adjuvant and antibody vaccines that specifically deliver antigenic targets to DCs.

## Background

Pathogen encounter by cells of the immune system represents a form of danger initially sensed by professional antigen presenting cells such as dendritic cells (DCs) that undergo specialization to prime naïve T and B lymphocytes leading to a cellular or humoral response or both [1-4]. There is substantial evidence that defined molecular events within DCs follow the biosynthesis of pro-inflammatory, inflammatory and anti-inflammatory cytokines/chemokines, notably the up-regulation of MHC Class I and II as well as co-stimulatory molecules (CD80 and CD86). These changes often promote the development of a potent effector T cell or antibody response needed to eradicate or contain pathogen-invaded tissue [5,6]. In recent years, several new studies have come forth that highlight the importance of Toll-like receptors (TLRs) and the critical role they play in integrating innate immunity with adaptive immunity [7,8]. These novel insights have provided the scientific and technological impetus for the burgeoning development and growth of a variety of strategies that are currently being pursued to target the TLRs either for inducing tolerance, enhancing immunity or even reversing autoimmunity [9-15]. Modulation of DCs ex vivo to achieve some of these goals is now highly plausible, resulting in a type of DC that can be effectively tailored to suit vaccine formulations [16]. There is also a better understanding of which TLRs to activate in combination and which to avoid [17-19]. In vivo, however, this task has remained a major challenge, presumably owing to poor targeting capabilities and the non-specific action of TLR activating ligands since similar TLRs also are expressed in non-antigen presenting cells [20,21]. Consequently, current strategies are limited to creating stable chemistries to conjugate these ligands to the vaccine of choice or by employing molecular engineering techniques to generate fusion protein products (e.g. studies in this laboratory), adenoviral or similar non-replicating vectors containing the antigen, CD40L or co-stimulatory molecules [11,22]. Recently, studies using the bacterial outer membrane protein A, such as KpOmpA (*Klebsiella sp*.-derived) or other bacteria-derived products have shown potent modulation of antigen presentation by DCs mediated via specific TLR molecules [23,24]. While the actions of these bacterial products and other TLR-specific ligands induce DC maturation, it must be recognized that not all modulating agents yield activated DCs which are required for the development of a classical Th1 immune response (CTL effector induction accompanied by IL-12p70, TNFα and IFNγ production) [reviewed in Ref. [8]]. Exploiting DCs for the purpose of delivering whole protein antigens while supporting TLR signaling might require that MR and particular TLRs be spatio-temporarily connected [25]. There is growing evidence from different laboratories establishing an association between TLRs and C-type lectin receptors (CLRs, such as, mannose receptor (MR), Dectin-1 and DC-SIGN among others) which can shape the outcome of the response depending on which TLRs and their adapters are assembled to interface with CLRs [21,26,27]. In this regard, the mannose receptor plays an important role in innate immune responses, especially in maintaining homeostasis and state of tolerance. However, we and others have successfully shown that endocytic receptors on DCs can be exploited to deliver exogenous soluble antigens for effective cross-presentation to T cells [28] using antibodies to MR [29-31], DEC-205 [32] DC-SIGN [33], Dectin-1 [34] and TLR2 [35-37]. While there is availability of synthetic or natural ligands for in vitro and in vivo studies in small animals, not all agents are suitable or adequately tested and formulated for human use. In the present study, we have investigated only those TLR activating agents which can be made as GMP material for combination with our vaccine candidate, B11-hCGβ.

The tumor-associated antigen, hCGβ, is overexpressed in a wide variety of human tumors including colorectal, pancreatic, bladder, renal, breast and germ cell tumors, among others [[31,38] and [39]]. Here, we show that DCs are modulated to prime T cell responses when the exposure to vaccine occurs in the presence of a TLR 3-specific dsRNA (poly I:C) and a TLR 7/8-specific agent, Resiquimod (R-848). The in vitro induction of primary cell-mediated immune responses to a normally non-antigenic protein in both normal donors as well as cancer patients points to DCs being the appropriate APCs for stimulation of the immune system. Thus the combination of TLR activation with an MR-targeted vaccine can significantly improve the therapeutic efficacy of DC-targeted vaccines.

## Materials and methods

### Human subjects

Peripheral blood from normal healthy volunteers was procured from a local blood bank (Biological Specialties Corp., Colmar, PA) or from pancreatic, bladder or colon cancer patients with informed consent following IRB approval from Duke University Medical Center, Durham, NC. The status of hCGβ expression in patient's blood or tumor was not determined in the patients for this study although such evidence has previously been documented in literature [38,39]. All blood products were handled under aseptic conditions. Leukapheresis of donors typically yielded 4–5 × 10^9 ^total cells, which were subsequently enriched for mononuclear cells using density gradient centrifugation. Cells from each donor were aliquoted at 5 × 10^7 ^– 1 × 10^8^/ml before cryopreservation in liquid nitrogen.

### Cell Lines

Two cell lines were used in determining the specificity of vaccine responses: T2, a TAP^-/- ^T-B hybrid cell line that is defective in antigen processing and K562, an HLA class I negative, NK-sensitive human eythroleukemia cell line (both obtained from ATCC, Manassas, VA). Both cell lines were maintained in IMDM + 10% FBS supplemented with 2 mM L-Glutamine and 25 μg/ml gentamicin.

### TLR agonists and MR targeted vaccines

The following TLR agonists were employed in experiments aimed at simultaneous engagement of TLRs and MR: R-848 (Resiquimod, 3M Pharmaceuticals, St. Paul, MN), a small molecule immunomodulator specific for human TLR 7 and 8. MALP-2 (TLRs 2, 6), Loxoribine (TLR7), Pam3CSK4 (TLR2), Flagellin (TLR5) all purchased from Invivogen (San Diego, CA), LPS^*E.coli *^(TLR4) and Poly I:C (TLR3) both purchased from Sigma-Aldrich (St. Louis, MO). CD40-specific CD40L-XL (crosslinked) was purchased from Alexis Biochemicals (Axxora Corporation, San Diego, CA).

The MR targeted vaccine is a fusion protein (designated B11-hCGβ) which consists of a fully human anti-MR mAb, B11 genetically fused, via its Fc portion, to the hCGβ subunit of human chorionic gonadotropin as previously described [30,31].

### Cytokines and mitogens

Polyclonal B and T cell activators- KLH and PHA, respectively, were obtained from Sigma-Aldrich Chemical Co. (St. Louis, MO). GM-CSF, IL-4 and IFNα were purchased from R&D Systems (Minneapolis, MN).

### Synthetic peptides

Two synthetic peptides of hCGβ used in this study correspond to previously characterized HLA-A2 restricted T cell epitopes derived from the following sequence: CPTMTRVLQGVLPAL [31]. The peptides TMTRVLQGV (hCGβ AA 60–68) and VLQGVLPAL(hCGβ AA 64–72) were custom made to >95% purity by Peptidogenic Research (Livermore, CA).

### HLA typing

HLA typing on peripheral blood lymphocytes was performed either by an HLA reference laboratory affiliated with the University of Colorado, Denver Health Sciences Center, Denver, CO (ClinImmune Labs; Aurora, CO) using a PCR based method or serologically using OneLambda's (Canoga Park, CA) HLA class I and class II loci typing trays with the resultant identity being confirmed by microcytotoxicity assay using ethidium bromide/acridine orange counterstaining. Normal donors (#s 4, through 8) and patients (#s 1 through 6) in this study typed HLA-A*0201.

### DC generation, hCGβ antigen uptake, activation and phenotyping

PBMCs were isolated from normal donor or cancer patient blood by density gradient centrifugation of enriched mononuclear cells. Immature DCs were prepared from PBMCs by incubating 2.5 × 10^6^/ml cells in T-75 flasks (Corning, NY) containing serum-free X-VIVO-15 medium (Cambrex, Walkersville, MD) to promote adherence of monocytes. Non-adherent cells were decanted and the adherent cells were washed three times with HBSS to remove platelets, B and T cells. Fresh X-VIVO-15 medium supplemented with GM-CSF (25 ng/ml) and IL-4 (50 ng/ml) was added to the adherent monocytes and on days 5–6 loosely adherent cells showing DC morphology (dendrite formation) were gently harvested after incubating the culture flasks at 4°C for 30 min. Detached DCs were washed three times in cold HBSS, resuspended at 1 × 10^6 ^cells/ml of serum-free AIM-V medium and treated with B11-hCGβ (10 μg/ml) for 30 minutes at 37°C. For DC maturation, either non-TLR (CD40L at 20 ng/ml) or TLR-specific agonists (2, 4, 5, 6, 7 and 8) were added to DC cultures with and without the MR targeted vaccine and incubated at 37°C for 16–18 hours. Cells were washed twice with cold PBS before staining with FITC or PE conjugated antibodies specific for CD80, CD83, CD86, HLA-DR and HLA-ABC (BD Biosciences, CA).

### Screening of DC activating agents by cytokine profiling and alloT cell responses

Monocyte derived immature DCs were generated from normal donor PBMCs by differentiation with IL-4 and GM-CSF as described in the previous section. DC maturation was assessed at two levels: (1) The upregulation of Th1 priming cytokines and (2) The induction of an MLR-type response. Such an assessment would indicate whether the agent was potent enough to drive DC-dependent proliferative T cell responses via enhanced expression of allo-HLA and co-stimulatory molecules on DCs. The concentration of specific TLR activators was used according to manufacturer's instructions. Monocyte-derived immature DCs (5 × 10^3^/well) were exposed to different TLR activators as single treatments with or without the vaccine at 37°C for 16–18 hours. Cell-free supernatants were harvested and analyzed in triplicate for a panel of cytokines and chemokines using both commercial kits (Proteoplex; EMD BioSciences, St. Louis, MO; Human Quant I/II, Schleicher-Schuell BioSciences/Whatman, Inc., Sanford, ME) and cytokine screening services (Allied Biotech, Human Cytokine Screening Service, (Ijamsville, MD). The concentration of each cytokine was expressed as pg/ml. Allogeneic T cell responses were evaluated by the degree of stimulation as measured by [^3^H]-thymidine ([^3^H]-TdR) incorporation using a β-scintillation counter. Responses of two fold higher relative to no stimulation controls were considered to be a positive response.

### In vitro generation of CTL and Granzyme B release from hCGβ-specific T cells

For in vitro stimulation of T cells, the non-adherent fraction of patient PBMCs (herein referred to as PBL) were co-cultured at 1.0 × 10^6 ^cells/ml/well, in 24-well plates with the antigen-exposed mature autologous DCs at 5 × 10^4 ^cells/ml/well (T cell: DC ratio = 20) for 3 to 4 consecutive weeks in AIM-V media. The cultures were supplemented with low doses of IL-7 and IL-2 (10 ng/ml each) in the first week and then maintained 20 ng/ml IL-2 to support the growth of antigen-activated T cells. Cytokines and media were changed every 3–4 days.

Five days after the second or third round of weekly re-stimulation, T cells were harvested and assessed in a cytotoxic Granzyme B release assay (GrB-ELISpot kit, Cell Sciences Inc., Canton MA; Ref. 40) as follows: Effector T cells from patient PBMCs were stimulated with either peptide pulsed T2 cells, antigen naïve DCs or treated autologous DCs at a 20:1 ratio directly in the wells of a Granzyme B (GrB) ELISpot plate for 15–20 hours at 37°C. The plates were washed and developed as per manufacturer's instructions (Cell Sciences Corp., Canton, MA). Spot formation was screened and evaluated by Zellnet Consulting, Inc. (Fort Lee, NJ). Results are reported as the number of GrB+ spots per 50,000 T cells in the well.

### Characterization of hCGβ-specific helper T cell responses

Effector T cells (5 × 10^4^/well) derived from patient PBMCs were stimulated at a 10:1 ratio in 96 well flat bottomed plates in 200 μl AIM-V medium with autologous DCs that were either antigen naïve or antigen-treated and matured with different TLR-activating agents. The cells were co-cultured for a total of 96 hours at 37°C and pulsed with [^3^H]-TdR during the last 18 hours (1 μCi/well, Amersham BioSciences, GE Healthcare, Piscataway, NJ) to measure uptake of thymidine. Cells were harvested and radioactivity was detected by scintillation counting (Wallac Microbeta Jet). Results are reported as mean ± S.D. of sextuplicate wells. Proliferation was also assessed by BrdU incorporation using a commercial ELISA kit (Roche Diagnostics, Nutley, NJ). Proliferation was evaluated using a Molecular Devices (Sunnyvale, CA) SpectraMax ELISA plate reader and SoftMax Pro software. To determine the stimulation index (S.I.), the "cpm" and "O.D." values (in the ^3^H-TdR assay and BrdU ELISA, respectively) from DC-vaccine-stimulated T cell cultures was divided by T cell cultures stimulated with DCs without vaccine treatment (control). An S.I. value greater than 2 fold higher than that in the control was considered a positive response.

### Statistical analysis

A two-tailed Student's t test for paired samples was used for comparison of vaccine only to vaccine plus adjuvant combination treatments using Microsoft Excel's statistical analysis tools for Windows NT. A 'p' value of 0.05 or less was considered to be significant.

## Results

### hCGβ vaccine-treated DC modulation with selected TLR-activating ligands

**Figure 1:**
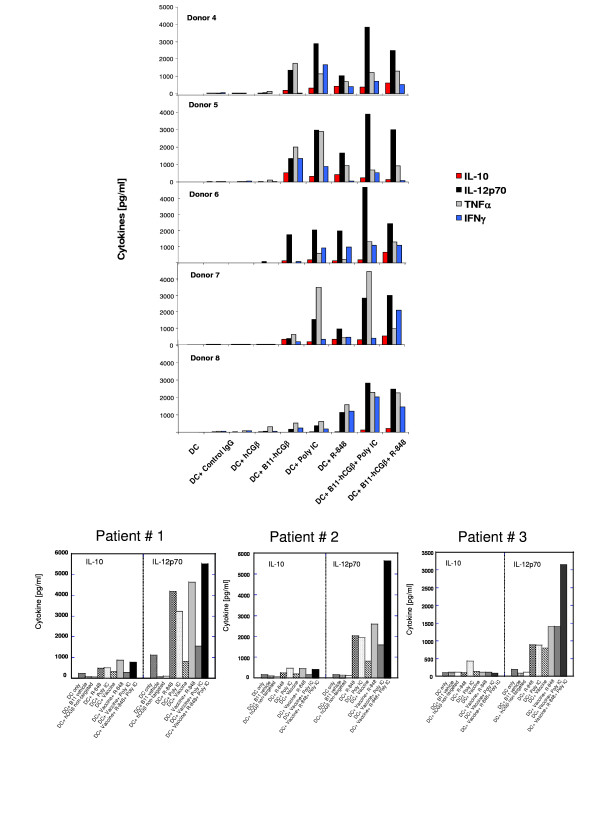
**Vaccine targeting mannose receptor polarizes dendritic cells to a Th1 cytokine phenotype**. Monocyte-derived DCs from normal donor PBMCs (1a) or cancer patients (1b) were treated as described under Methods. (1a) Differential Th1-type cytokine production by DCs treated separately with vaccine (B11-hCGβ at 10 μg/ml) and TLR activating agents; (1b) Vaccine-specific DC-dependent Th1 cytokine induction in cancer patients.

Monocyte-derived DCs were harvested on day 5 and exposed to every combination of the vaccine (B11-hCGβ) in conjunction with the ligands specific for the various TLR (2, 4, 5, 6, 7 and 8) or non-TLR molecules (CD40, IFN receptor). First, we examined several normal donor DC samples to optimize experimental conditions which included a 30 minute exposure to the vaccine at 37°C followed by the addition of TLR-specific ligands to DC cultures for further incubation (~18 hours). Cell-free supernatants were collected thereafter and analyzed by cytokine multiplex arrays. As shown in Fig. 1a (donor #s 4–8), IL-12p70 produced by DCs in response to adjuvants poly I:C and R-848 in combination with the vaccine (B11-hCGβ) was enhanced compared to poly I:C or R-848 treatment alone. Interestingly, DCs from donor #6 showed equivalent IL-12p70 production DCs treated with vaccine alone or adjuvant alone and vaccine+ R-848 combination. R-848 + vaccine treatment of DCs markedly enhanced IL-12p70 production compared to R-848 treatment alone in donor #s 4, 5, 7, 8 whereas poly I:C+ vaccine treated DCs produced IL-12p70 more than poly I:C alone in donors 6, 7 and 8. In all donors tested the relative levels of IL-10 in the vaccine + poly I:C or vaccine+ R-848 was 3–10 fold lower compared to that of IL-12p70. E

The possibility that an underlying cross-talk between TLR and MR signaling pathways might contribute towards a Th1 T cell polarizing event prompted us to examine the effect of independently engaging different TLR agonists in the presence of B11-hCGβ vaccine. As shown in Table 1, DCs from donor #4 and #5 were considered for extended TLR-MR activation studies. Thus, DCs that were treated with TLR-specific ligands with or without the vaccine were assessed for two opposing cytokines that are representative of a Th1 response (IL-12p70, a critical regulator of adaptive immune responses) or a Th2 response (IL-10, an anti-inflammatory cytokine). DCs were screened for a cytokine response to various stimuli directed at TLR (2, 3, 4, 5, 6, 7 and 8) and non TLR receptors (CD40, IFN). Data represented in Table 1 show that the vaccine in combination with activation signals is consistently contributing to a Th1 type response (IL-12p70 > IL-10). In the case of TLR2 stimulation, however, IL-12p70 production is dampened when MALP-2, but not Pam3CSK4, is used as the ligand for this receptor suggesting that additional TLRs (presumably, TLR6) may be involved in attenuating the TLR2-dependent signaling. For the two adjuvants that appear to be potentially applicable in the clinic, namely poly I:C and R-848, combination with vaccine clearly augmented DC-dependent IL-12p70 production compared to IL-10 suggesting a Th1-bias (p < 0.01 and p < 0.05, respectively). Signaling via TLR-independent pathways such as those obtained by CD40-CD40L interaction further indicate that antibody targeting of MR may intersect with MAP kinase pathways via common downstream adaptor molecules which are yet to be characterized.

**Table 1:** Production of Th1-like cytokines by activated DCs

**Immature Mo-DC Treatment**	**Donor 4**				**Donor 5**			
	
	**No vaccine**	**Vaccine**	**No vaccine**	**Vaccine**	**No vaccine**	**Vaccine**	**No vaccine**	**Vaccine**
	**IL-10**	**IL-10**	**IL-12p70**	**IL-12p70**	**IL-10**	**IL-10**	**IL-12p70**	**IL-12p70**
	
None	43 ± 2	61 ± 10	145 ± 10	498 ± 23	30 ± 4	23 ± 10	115 ± 18	121 ± 11
Activated CD4+ T	46 ± 8	149 ± 3	189 ± 16	154 ± 12	60 ± 15	241 ± 3	234 ± 6	644 ± 6
CD40L XL	45 ± 3	211 ± 21	185 ± 40	4818 ± 18	136 ± 11	513 ± 9	1139 ± 24	1406 ± 6
LPS ^*E.coli*^	171 ± 16	1041 ± 26	308 ± 11	1588 ± 1 10	415 ± 4	552 ± 11	1089 ± 19	1859 ± 60
Poly I:C *	184 ± 11	307 ± 6	**2221 ± 210**	**8327 ± 240**	308 ± 10	475 ± 24	**1369 ± 58**	**2847 ± 36**
MALP -2	87 ± 13	125 ± 12	7646 ± 110	169 ± 33	41 ± 12	574 ± 22	1512 ± 26	610 ± 11
Pam3CSK4	28 ± 4	81 ± 11	40 ± 7	430 ± 23	441 ± 22	691 ± 31	644 ± 15	797 ± 18
Flagellin	54 ± 6	26 ± 7	80 ± 30	335 ± 18	1211 ± 20	1406 ± 11	809 ± 11	4110 ± 51
Loxoribine	22 ± 11	72 ± 8	34 ± 16	1141 ± 9	691 ± 13	1106 ± 31	1839 ± 15	4293 ± 15
R-848 **	12 ± 3	1106 ± 21	**43 ± 10**	**4293 ± 228**	667 ± 9	823 ± 11	**983 ± 21**	**2103 ± 121**

### TLR activation also induces potent Th1 cytokine production in patient DCs

To investigate whether the Th1-type modulation observed above in normal donors (Fig. 1a) can be reproduced in cancer patients, monocyte-derived DCs from a subset of cancer patient PBMCs were treated with vaccine in the absence or presence of TLR agonists. Monocyte-derived DCs from all three patient demonstrated that activated TLRs can enhance IL-12p70 cytokine production in concert with MR-targeting. Moreover, these responses appear to be highly vaccine-specific (i.e. directed to hCGβ component of the vaccine) since controls (vaccine-naïve DCs or DCs exposed to a non-targeted soluble hCGβ), the vaccine vehicle (B11) and a isotype-matched control IgG were not able to induce Th1 cytokines (Fig. 1b). In addition, DC modulation with poly I:C and R-848 in the absence of vaccine targeting can still induce IL-12p70, but the secretion was enhanced with vaccine combined with TLR activation during DC stimulation. Also, as noted for normal donor DCs, the effect mediated by vaccine only treated DCs was further augmented by the addition of TLR 3, 7/8 activation signals. As in normal donor DCs, IL-10 production by patient DCs was several fold lower compared to IL-12p70, indicating a dominant polarization to a Th1 type DC.

### Induction of allogeneic response following MR and TLR targeting of DCs

**Figure 2:**
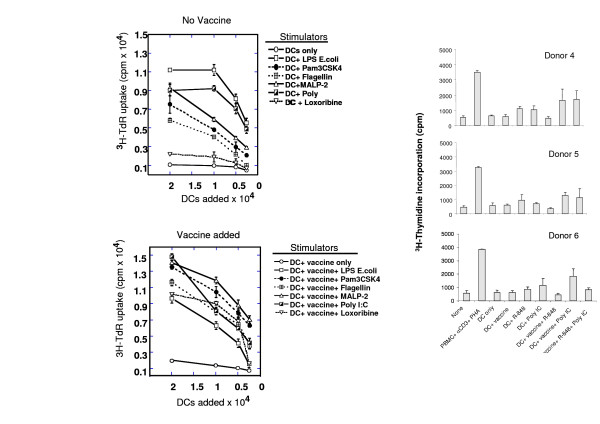
**Induction of alloresponses by MR-targeted vaccine combined with TLR activation**. DCs from normal donor PBMCs were untreated (2a, 2c) or treated (2b, 2c) with vaccine (B11-hCGβ at 10 μg/ml) in combination with TLR activating ligands and added to allogeneic T cells post incubation at different DC: T cell ratios. T cells were pulsed with [^3^H]-TdR on day 4 and harvested on day 5 to assess incorporation of DNA-associated radioactivity. Experiments were performed in triplicates. Data are shown as mean ± S.D. of 2 donor (2a, 2b) and 3 donor samples (2c), respectively.

In an allogeneic mixed lymphocyte reaction assay, T cells respond by proliferation to changes in the level of MHC class I, II and co-stimulatory molecules on the stimulatory DCs regardless of the antigen being presented. Using normal donor PBMCs, T cells from one donor were co-cultured with treated DCs from a different donor (HLA-mismatched at several loci) for 5 days and pulse chased for the last 18 hours with tritiated thymidine (^3^H-TdR). As depicted in Fig. 2, strong proliferative responses were noted for a variety of DC treatments as shown by increased uptake of thymidine. However, DCs that received TLR signaling in the absence of antigen (no vaccine) did not induce T cell proliferation to the same extent (Fig. 2a) as those that were exposed to the vaccine (Fig. 2b). The differential between the two treatments was apparent in the case of TLR 2, TLR3, TLR 5 and TLR 7-specific activation suggesting that the vaccine clearly contributes to the response. Surprisingly, the TLR 7/8 agonist, R-848-mediated modulation of DCs did not induce a strong proliferative alloresponse with or without vaccine (Fig. 2c) suggesting that TLRs 7 and 8 expression may be reduced in monocyte-derived DCs. Stimulation indices calculated for the different stimuli, as shown in Table 2, further shows that most TLR-specific stimuli, with the exception of LPS and R-848 as single agents and R-848 combined with poly I:C, were able to elicit a detectable allogeneic response when combined with B11-hCGβ vaccine.

**Table 2:** Induction of Allogeneic T cell responses by DC modulating agents

DC Treatment	TLR specificity	No Vaccine S.I.	Plus Vaccine S.I.
None		-	-
+ LPS	4	4.1	4.0
+ Pam3CSK4	2	3.0	5.6
+ Flagellin	5	2.4	4.8
+ MALP-2	2, 6	3.0	5.6
+ Poly IC	3		
(Fig. 2a-2b)		3.6	6.0
Fig. 2c		2.4	3.8
+ Loxoribine	7	1.25	4.0
+ R-848	7, 8	2.39	1.85
(Fig. 2c)			
+ Poly IC + R-848	3, 7, 8	NT	1.95
(Fig. 2c)			

NT-not tested; Stimulation indices derived from allogeneicT cell responses in a standard 5-day ^3^H-TdR incorporation assay. Two donors were tested in Figure 2a & 2b and three donors were tested in Figure 2c.

### R-848 can modulate DC surface phenotype independent of antigen

The availability of a clinical-grade class of imiquimod, R-848, that specifically activates TLR7/8 expressing cells such as human DCs, prompted us to investigate the properties of this immunomodulatory agent in the context of MR targeting. Immature monocyte-derived DCs were treated with a range of doses of R-848, with or without the B11-hCGβ vaccine for 24 or 48 hours [see Additional files 1 and 2] and stained for maturation/activation markers CD80, CD83, CD86, HLA-ABC and HLA-DR. The upregulation of DC HLA-DR expression following R-848 treatment (MFI 1807 treated vs. 1324 untreated), CD80 (MFI 447 treated vs. 256 untreated) and CD86 (MFI 447 treated vs. 265 untreated) was noted with R-848 with or without the vaccine. In contrast, the expression of HLA-ABC only marginally changed (MFI 186 treated vs. 171 untreated) while levels of CD83 did not appear to be particularly affected by the treatments (MFI 25 treated vs. 24 untreated). These results indicate that DC surface markers can be sufficiently modulated with R-848 to prime T cell function which subsequently led to studies on the induction of autologous T cell responses in patients. The upregulation of HLA and co-stimulatory markers on DCs using other TLR activating agents in combination with B11 mAb has been observed using LPS or Flagellin (unpublished observations). While the latter observation is consistent with alloresponses, the lack of T cell effector function in an autologous setting suggests that signaling via TLR4 or TLR5 in conjunction with MR signaling may not adequately support Th1 priming as that generally seen with CD40-CD40L or TLR3 or TLR 7/8 signaling (IL-12p70 induction).

### In vitro CTL induction in cancer patients

**Figure 3:**
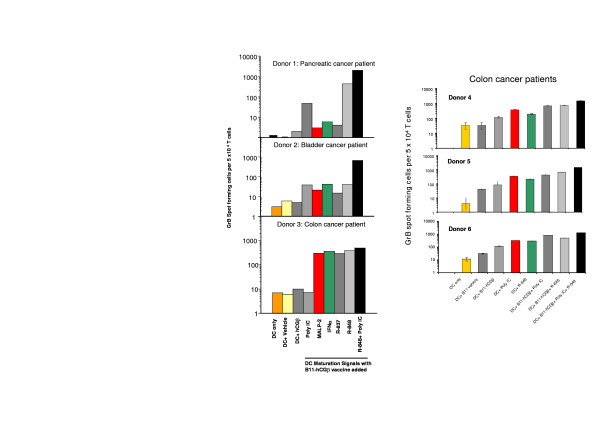
**In vitro induction of CTL responses in cancer patient PBMCs**. T cells were stimulated with autologous DCs treated as described under Methods. Briefly, stimulations were performed once every 8 days for a total of 4 weeks with cytokine additions- IL-2 and IL-7 (10 ng/ml each) and expanded after the third stimulation using anti-CD3/anti-CD28 beads. Expanded cells were harvested on day 14 and restimulated with DC+ vaccine (B11-hCGβ, 10 μg/ml) followed by activation with different stimuli (3a-c donor #s 1–3) or R-848 (0.1 μM or 0.3 μg/ml.) and Poly IC (50 μg/ml) added alone or in combination (3d, donor #s 4–6). T cells were assayed for effector function on days 5 and 6 post stimulation using the Granzyme B ELISpot assay kit.

Since DCs appear to be amenable to modulation with TLR-activating ligands, it was important to determine whether antigen-specific cytolytic effector T cells could be induced in vitro in primary cultures of autologous peripheral blood lymphocytes (PBLs) isolated from cancer patients. DCs were generated from three HLA-A2+ cancer patients with different cancers (pancreatic, bladder and colon) and treated as before (vaccine+ R-848+ poly I:C). The stimulated DCs were co-cultured with autologous PBLs for 3–4 weeks in the presence of IL-7 and IL-2 as indicated in the *Materials and Methods *section. Effector CTL detection was assessed in a Granzyme-B ELISpot assay against a panel of DC targets with a T cell: DC ratio of 20:1. As shown in Figs. 3a–3d, sensitization of pancreatic and bladder cancer patient PBMCs to the vaccine-adjuvant combination generated higher numbers of vaccine specific T cells responding to DCs presenting the cognate form of the vaccine but not to controls (DC, DC-vehicle or DC-soluble hCGβ). The effector response in the PBMCs of a colon cancer patient (# 3), however, yielded a broader response to DC targets modulated by different TLR activators. Additional patients tested (#s 4, 5 and 6) showed increased CTL responses when stimulated by DCs exposed to vaccine in combination with both poly I:C and R-848 compared to vaccine and either agent alone. Overall, the data indicate that DC-vaccine can be optimally modulated with the addition of TL3 and TLR 7/8 activating ligands (Fig. 3d).

### Enhanced epitope-specific CTL response

**Figure 4:**
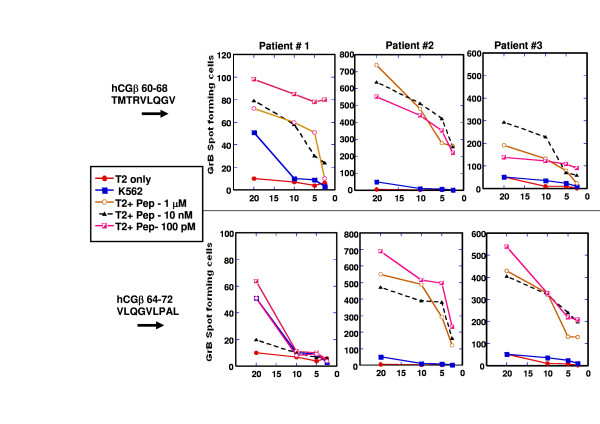
**Cytolytic T cell response to vaccine in patient PBMCs includes a high affinity T cell compartment with specificity for two HLA-A2-restricted hCGβ-derived epitopes**. Granzyme B-ELISpot determination of antigen specific T cells in three cancer patients shows significant effector function is mediated by CD8+ T cells compared to NK cell activity (K562 lysis). T cells were assessed for activity in triplicates using peptide-loaded TAP -/- T2 cells in the presence of 3 μg/ml β 2 m in AIM-V medium for 3 hours at 37°C before transfer to GrB ELISpot plates for a further 16 hour incubation and processed as described under Methods.

To ascertain the true specificity of vaccine-specific responses in the foregoing experiments, two HLA-A2-binding peptides derived from the hCGβ sequence were identified (hCGβ 60–68: TMTRVLQGV and hCGβ 64–72: VLQGVLPAL). Synthetic peptides were loaded at different concentrations onto a TAP^-/- ^HLA-A2+ T2 cell line which served as targets. CTL generated in vitro from patient PBMCs as described before were tested at different effector to target ratios against peptide-pulsed or non-pulsed T2 targets with K562 cells serving as an HLA class I negative control. Whereas cytolytic T cells were induced in all three patients, patients #2 and #3, in particular, recognized both epitopes equally well while patient #1 responded less well relative to controls (Fig. 4). CTL thus generated appeared to be of high affinity since sub-nanomolar concentrations of the peptide were sufficient to sensitize T2 targets to CTL-mediated lysis. Enumeration of vaccine-specific T cells revealed that DC-vaccine combination with TLR3 and TLR7/8 activation using R-848 and poly I:C preferentially expanded a higher frequency of CTL precursors (CTLp) in all three patients than R-848 alone. T cell effector generation with either TLR agent (poly I:C or R-848) in combination with the vaccine resulted in AICD (activation-induced cell death) of T cells over a period of several weeks (usually after second or third re-stimulation). In terms of CTLp frequency with specificity for hCGβ HLA class I-restricted epitopes presented by non-professional APCs (autologous PBMCs), responses were only noted in patient #3 [hCGβ 60–68: 1/400 and hCGβ 64–72: 1/316; see Additional file 3]. The observed variation in the frequency of GrB+ CTL vis-à-vis antigen presentation by vaccine-pulsed (DCs) or peptide-pulsed APCs (PBMCs) may be related to DC presentation of multiple (unknown) epitopes that could contain post-translational modifications, as also to additional signals required for complete effector function of CTL only mediated by DCs, but not PBMCs. These results indicate that CTL not only recognize whole antigen processed and presented by DC HLA class I molecules but also a pre-processed form of the antigen presented by alternate APCs (PBMCs or T2 cells) thus by-passing antigen processing requirements.

### Induction of hCGβ-specific T helper responses

**Figure 5:**
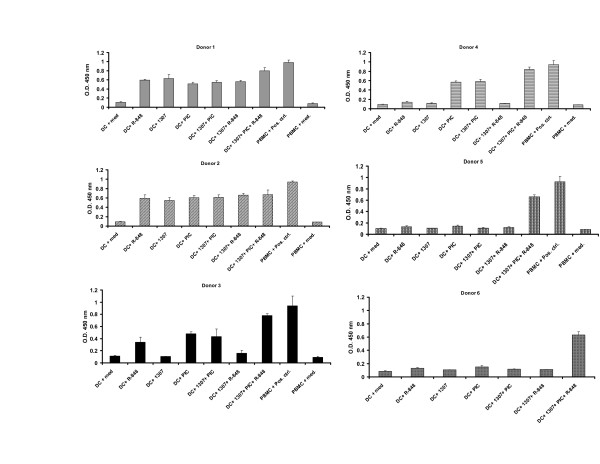
**Antigen-specific T helper response is amplified when vaccine-treated DCs are further modulated with R-848 and Poly I:C**. T cells were processed as described in the legend to Fig. 3. Briefly, responder T cells from six cancer patients and autologous DCs were co-cultured at a ratio of 10:1 for 5 days in sextuplicates. Cultures were pulsed with BrdU reagent during the last 16 hours after which DNA synthesis was measured by assessing BrdU uptake with a BrdU ELISA kit.

The stimulatory capacity of vaccine-treated patient DCs modulated by TLRs 3 and 7/8 ligands was next assessed for induction of helper T lymphocyte (HTL)-dependent responses in a BrdU incorporation assay. As shown in Fig. 5, effector T lymphocytes from all 6 patients co-cultured with autologous DCs modulated as described above appear to proliferate extensively not only when presented with DC stimulators in a cognate form (R-848+ Poly I:C) but also when R-848 alone was used for modulation. Overall the results show that R-848 was not superior to poly I:C for any donor. In 2/6 donors (#s 1 and 2) the two agents were equivalent in their immunodulatory effects on DCs. In other donors (#s 3 and 4), poly I:C was only slightly better than R-848. For 2/6 donors (#s 5 and 6) neither adjuvant worked as a single agent. (p < 0.05). The observed differences in DC response patterns between the donors are not unexpected due to genetic variability between individuals. Despite these variabilities two notable observations can be made. First, particular response patterns were repeated in unrelated donors (1 and 2; 3 and 4; 5 and 6) and second, it can be seen that for 6/6 donors the combination of R-848 and poly I:C was superior to single agent treatment suggesting the possibility of identifying a vaccine and adjuvant combination that can effectively stimulate DCs in spite of immune system polymorphisms. Stimulation indices determined in the different donors for their respective treatments also appear to be consistent with the above findings (Table 3).

**Table 3:** Autologous T helper responses to vaccines in cancer patients

Stimulation index						
DC treatment	Donor 1	Donor 2	Donor 3	Donor 4	Donor 5	Donor 6

Vaccine only	6	5.7	0.0	0.0	0.0	0.0
R-848 only	5.3	5.9	3.6	0.5	0.5	0.5
Vaccine + R -848	5.8	6.2	0.5	0.0	0.0	0.0
Poly IC only	5	5.9	5	5.8	0.0	0.6
Vaccine + Poly IC	5.8	6.0	4.8	6.0	0.0	0.0
Vaccine + R -848 + Poly IC	9.0	6.2	8.0	9.0	6.5	7.0

T cell lines were derived from 6 different donors (all cancer patients) and generated by in vitro stimulation with autologous DCs. Briefly, DCs were first exposed to vaccine (B11-hCGβ) and further activated with R-848 and Poly I:C and T cells added to the mix. Proliferative responses were tested in a standard 5-day BrdU incorporation ELISA. Values are relative to unstimulated DCs.

## Discussion

Despite the fact that dendritic cells have been largely manipulated ex vivo for use as antigen-presenting vehicles to deliver peptide vaccines, the approach is restricted to patients with a desired profile such as the most frequent HLA type and by the limited number of peptides that can be used to stably associate with these HLA molecules. To make DC-based vaccines amenable to a wider cross-section of the patient population, vaccines could be designed to include a whole protein that can be delivered directly in vivo with high DC-specific targeting properties. This strategy will not only overcome the current limitation of HLA type of eligible patients but will also circumvent the need to identify peptides that bind HLA with high affinity and eliminate costly DC manipulations *ex vivo*. Vaccines can be made more efficacious by providing DC activation signals in vivo (i.e. adjuvants), an effect that is necessary to drive a desired immune response. However, since DCs do exist in different forms (subsets) with varying composition of Toll-like receptors [17], it would be almost necessary to activate only those DC subsets that bear the appropriate vaccine and adjuvant receptors (e.g. MR and TLR). Currently, several approaches are being taken to identify such adjuvant-vaccine combinations. Given the challenges that patients bring to the clinic including compromised sample quality and quantity, the notion of conducting large scale healthy volunteer in vitro studies and model systems such as the use of HEK-293 TLR or MR transfectants cell lines to mimic the phenomena in patients may not readily resolve the pressing issue of finding the right fit between antibody vaccines and TLR-specific adjuvants.

In this study we have screened commercially available synthetic ligands as well as ligands purified from natural sources to trigger selected TLR molecules. Specifically, our goal was to exploit agents for DC modulation that would be compatible with antibody-targeted vaccines and be readily translated to the clinic. To this end, we have selected monocyte-derived DCs for two reasons- (a) they most closely resemble their counterparts in the dermis and the interstitial tissues in terms of their immature state and expression of MR [39]; and (b) dermis is the route of administration in an ongoing Phase I study of the B11-hCGβ vaccine. In addition, proof-of-concept studies in our laboratory have previously shown that targeting MR on DCs with human antibodies is an efficient way to deliver tumor antigens to the HLA class I and II- antigen presentation pathway for activation of T cells restricted by these molecules [30,31]. Other studies also have reported the efficiency of targeting antigens to different C-type lectins on DCs such as DEC-205 [32] and DC-SIGN [33] with antibodies and have identified DC activation signals needed to overcome tolerance.

The experimental strategy was designed to assess the immune modulation of DCs at three levels- (a) phenotypic maturation, i.e. to document changes in surface expression of activation markers such as HLA, co-stimulatory and differentiation antigens; (b) functional maturation, i.e. measuring levels of cytokine secretion using conditions established in previous studies with demonstration of an allogeneic T cell response; and (c) induction of effector T cell-mediated immune response, i.e. generation of antigen-specific CTL and helper T lymphocytes (HTL). Initial attempts at T cell cultures with the different TLR activators combined with our mAb vaccine (B11-hCGβ) revealed that poly I:C and R-848 were the only agents that were capable of stimulating short-term T cell cultures. The reasons for the failure of other TLR activators is not apparent but could be related to the quality of induced maturation/activation of DCs or tolerizing functions of cytokines per se [42,43]. In addition, studies were performed with dual TLR activators -poly I:C and R-848 in combination with vaccine both in normal donors and patients. At all three levels of assessment, our data satisfy the above criteria as an optimal way to activate DCs for effective stimulation of T cells.

In summary, the preferential secretion of Th1-biasing cytokine IL-12p70 over IL-10, the increase in CTL precursor frequency that is both vaccine- and epitope-specific and an HTL response with similar specificity strongly suggests that TLRs and MRs may be interacting. Of note is the observation that MR targeting in the absence of additional (TLR) signals directly contributes to IL-12p70 and this response is heightened when poly I:C is added to the mix and vice versa. R-848 alone can induce IL-12p70 in DCs but unlike TLR3, which is intimately connected with NF-κB signaling pathway, R-848 may exert its effect primarily via type I IFN secretion which, in turn, activates IL-12 genes [44]. Induction of a heightened allogeneic by DCs has remained the hallmark of activation state of DCs. In this regard, it was interesting to note that unlike the previously reported immunomodulatory properties [45], the use of R-848 its use in the context of our vaccine did not produce an allogeneic T cell response, presumably owing to differences in DC generation methods (day 7 or day 8 DCs compared to day 5 or day 6 DCs in this study), higher concentration of R-848 (8 μg/ml vs. 0.3 μg/ml) and the use of purified allogeneic T cells. However, in an autologous setting, the induction of cytokine granule secretion by CTL was evident (Figs. 3 and 4). Although the direct modulation of DCs in vivo with R-848 has not been investigated, the possibility that bystander cells that express TLRs 7 and 8 could be activated to act in concert with DCs and Mös and to augment T cell cross-priming [46].

An interesting and important question is whether there is co-localization of the MR and TLRs in the endocytic pathway. There is preliminary evidence from co-localization studies showing that MR was found in association only with TLR3, but not TLR4, TLR7 or TLR8 (unpublished observations). It is unclear why there was no detectable TLR4 staining in mature DCs but it could be related to the DC maturation pathway [47]. In addition, the activation of DCs via TLR9 was not pursued in this study since, in humans, dermal and monocyte-derived DCs do not express TLR9. Conversely, DCs that express TLR9, such as plasmacytoid DCs (pDCs) do not express MR and therefore, were not suitable for targeting.

In general, the idea of engaging specific TLRs is beneficial both to augmenting antigen presenting capacity of DCs as well as sustaining a high avidity effector T cell repertoire. Although all of the above data consistently show immunopotentiating effects of TLR ligands in augmenting immunological responses to an MR-targeted vaccine, it is not certain whether engagement of two independent TLRs is a requirement in vivo (in human trials). It is important to consider that the vaccine product would now have three different entities requiring additional regulatory hurdles to overcome so as to secure approval for use in humans. Another consideration is that the inclusion of TLR activators in vaccine formulations is limited in that individuals vary in their TLRs owing to genetic polymorphisms. This is the case with TLR2 and TLR4 where mutations have been reported [48]. Therefore, caution must be exercised in deciding which TLRs must be activated since individuals with TLR deficiencies cannot respond to danger signals [49].

## Competing interests

VR, JPV, MAB and TK are employed by the industry; JDT and PKW declare no competing financial interests.

## Authors' contributions

VR and TK conceptualized the targeting technology and conducted antigen presentation assays and in vitro human T cell response analysis described in the present study in collaboration with PKW and JDT; JPV contributed to DC modulation experiments with R-848 and related analogs developed at 3M Pharmaceuticals using flow cytometry; and MAB essentially carried out multiplex cytokine analysis, GrB ELISpot and BrdU assays.

## Supplementary Material

Additional File 1 Donor DC dose response to R-848 in the presence and absence of vaccine. Click here for file

Additional File 2 Phenotyping of DC surface markers following exposure to vaccine in combination with TLR7/8 agonist R-848. Click here for file

Additional File 3 Enumeration of hCGβ antigen-specific CTLp frequency by GrB ELISPot assay. Click here for file
