# Supplementary material for: Toll-like receptor activation enhances cell-mediated immunity induced by an antibody vaccine targeting human dendritic cells
Source: J Transl Med. 2007 Jan 25;5:5. doi: 10.1186/1479-5876-5-5 (PMC1794405; doi:10.1186/1479-5876-5-5)
Supplement: Additional File 1 — Donor DC dose response to R-848 in the presence and absence of vaccine. [file 1479-5876-5-5-S1.ppt]

## Slide 1
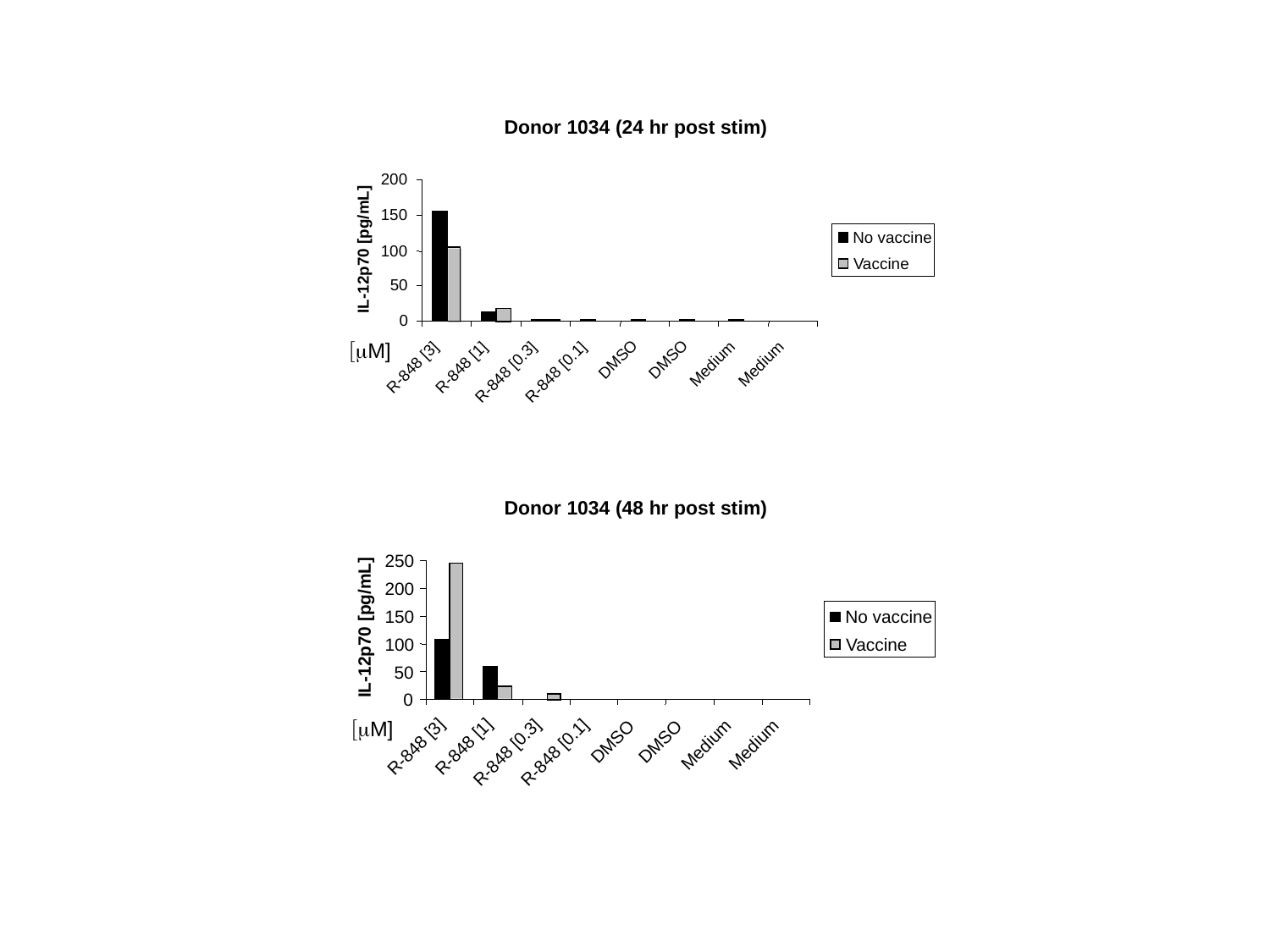

Donor 1034 (24 hr post stim)
200
150
No vaccine
IL-12p70 [pg/mL]
100
Vaccine
50
0
M]
DMSO
DMSO
Medium
Medium
R-848 [3]
R-848 [1]
R-848 [0.3]
R-848 [0.1]
Donor 1034 (48 hr post stim)
250
200
150
No vaccine
IL-12p70 [pg/mL]
100
Vaccine
50
0
M]
DMSO
DMSO
Medium
Medium
R-848 [3]
R-848 [1]
R-848 [0.3]
R-848 [0.1]
