# Supplementary material for: Toll-like receptor activation enhances cell-mediated immunity induced by an antibody vaccine targeting human dendritic cells
Source: J Transl Med. 2007 Jan 25;5:5. doi: 10.1186/1479-5876-5-5 (PMC1794405; doi:10.1186/1479-5876-5-5)
Supplement: Additional File 2 — Phenotyping of DC surface markers following exposure to vaccine in combination with TLR7/8 agonist R-848. [file 1479-5876-5-5-S2.ppt]

## Slide 1
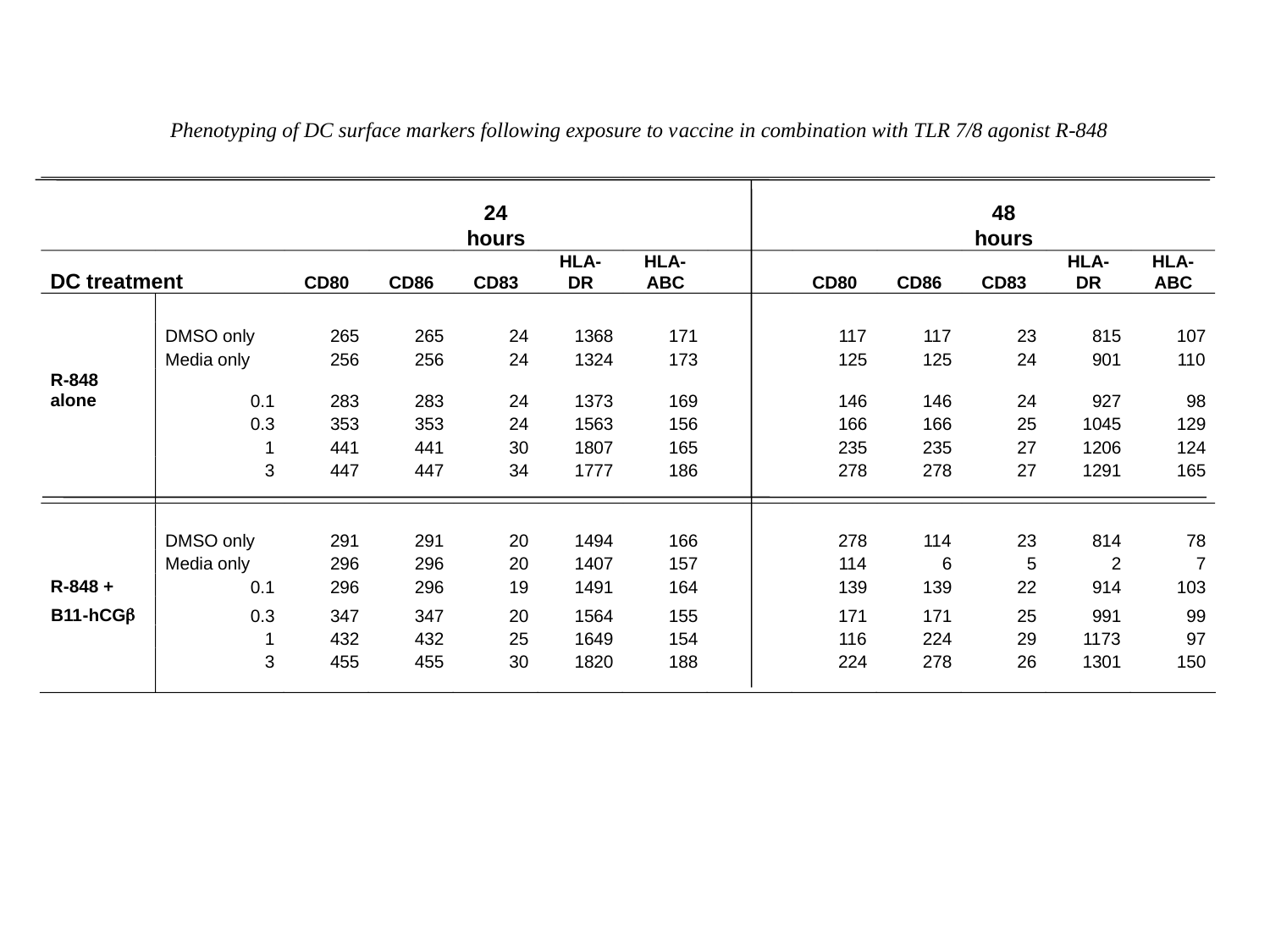

Phenotyping of DC surface markers following exposure to v
accine in combination with TLR 7/8 agonist R
-
848
24
48
hours
hours
HLA
-
HLA
-
HLA
-
HLA
-
DC treatment
CD80
CD86
CD83
DR
ABC
CD80
CD86
CD83
DR
ABC
DMSO only
265
265
24
1368
171
117
117
23
815
107
Media only
256
256
24
1324
173
125
125
24
901
110
R
-
848
alone
0.1
283
283
24
1373
169
146
146
24
927
98
0.3
353
353
24
1563
156
166
166
25
1045
129
1
441
441
30
1807
165
235
235
27
1206
124
3
447
447
34
1777
186
278
278
27
1291
165
DMSO only
291
291
20
1494
166
2
78
114
23
814
78
Media only
296
296
20
1407
157
114
6
5
2
7
R
-
848 +
0.1
296
296
19
1491
164
139
139
22
914
103
β
B11
-
hCG
0.3
347
347
20
1564
155
171
171
25
991
99
1
432
432
25
1649
154
116
224
29
1173
97
3
455
455
30
1820
188
224
278
26
1301
150
