# Supplementary figures and images for: Toll-like receptor activation enhances cell-mediated immunity induced by an antibody vaccine targeting human dendritic cells
Source: J Transl Med. 2007 Jan 25;5:5. doi: 10.1186/1479-5876-5-5 (PMC1794405; doi:10.1186/1479-5876-5-5)

## Slide 1
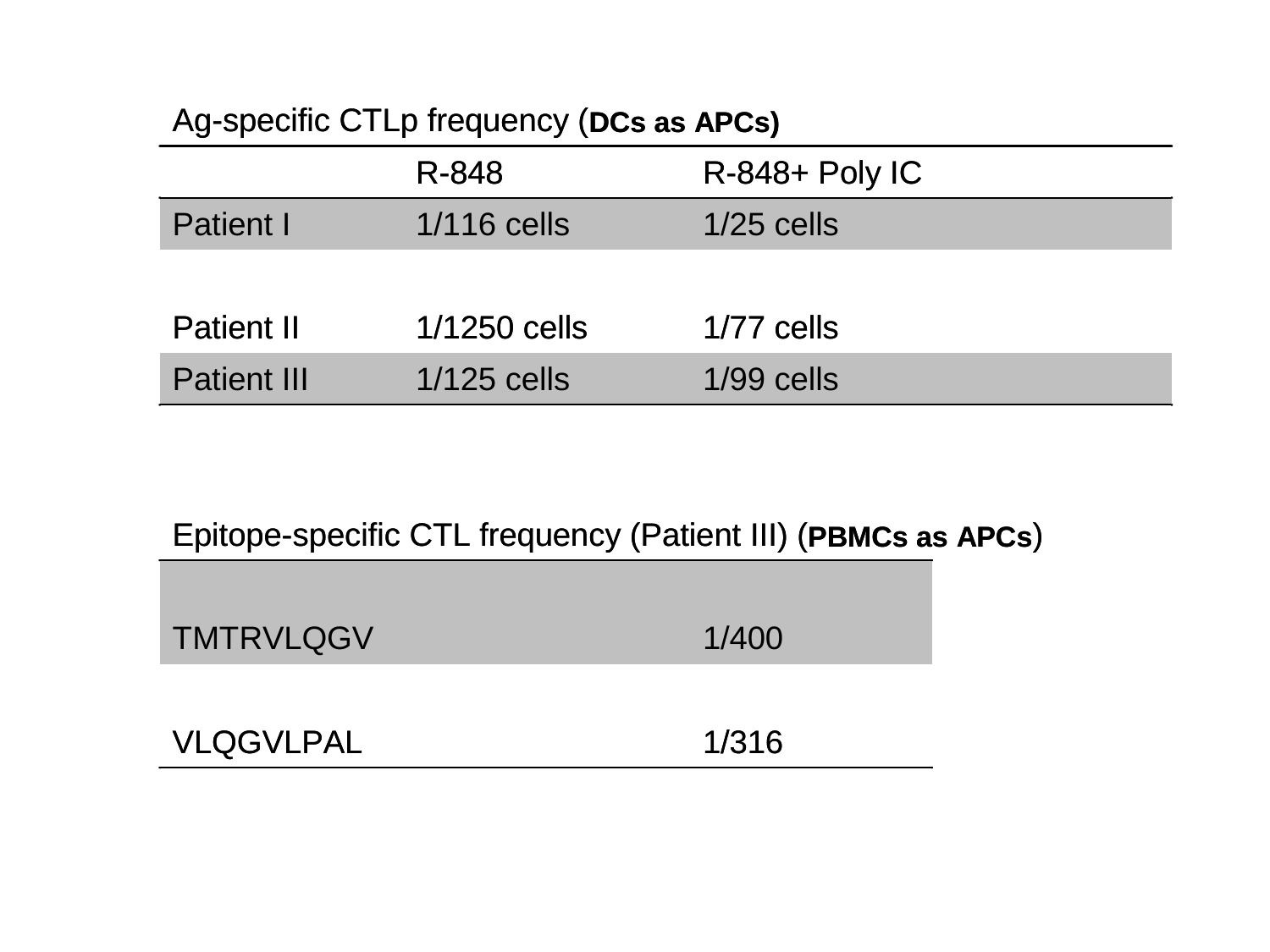

Supplement: Additional File 3 — Enumeration of hCGβ antigen-specific CTLp frequency by GrB ELISPot assay. [file 1479-5876-5-5-S3.ppt]
